# Knowledge, attitude, and practice of atrial fibrillation in high altitude areas

**DOI:** 10.3389/fpubh.2024.1322366

**Published:** 2024-04-10

**Authors:** Ke Li, Jinfeng Liu, Yan Zhu

**Affiliations:** ^1^Department of Cardiovascular Medicine, 363 Hospital, Wuhou, China; ^2^Department of Cardiovascular Medicine, Chengdu Fifth People’s Hospital, Wenjiang, China

**Keywords:** atrial fibrillation, high altitude, general population, knowledge, attitude, practice

## Abstract

**Background:**

To investigate the knowledge, attitude, and practice (KAP) of atrial fibrillation (AF) among the general population in high-altitude areas.

**Methodology:**

A web-based cross-sectional study was conducted among the general population in high-altitude areas.

**Results:**

A total of 786 valid questionnaires were enrolled, with a mean age of 34.75 ± 14.16 years. The mean score of knowledge, attitude and practice were 8.22 ± 6.50 (possible range: 0–10), 28.90 ± 5.63 (possible range: 8–40), 34.34 ± 6.44 (possible range: 9–45), respectively. The multivariate analysis showed that knowledge scores (OR = 1.108, 95% CI = 1.075–1.142, *p* < 0.001), attitude scores (OR = 1.118, 95% CI = 1.081–1.156, *p* < 0.001), and never smoking (OR = 2.438, 95% CI = 1.426–4.167, *p* = 0.001) were independently associated with proactive practice. The structural equation modeling (SEM) showed direct effect of knowledge on practice (*p* = 0.014), and attitude on practice (*p* = 0.004), while no effect of knowledge on attitude (*p* = 0.190).

**Conclusion:**

The general population in high-altitude regions had adequate knowledge, positive attitude, and proactive practice towards AF. The SEM was suitable for explaining general population’ KAP regarding AF, revealing that knowledge directly and positively affected attitude and practice.

## Introduction

Atrial fibrillation (AF) stands as the predominant arrhythmia encountered in clinical practice. Its global prevalence exceeds 33 million individuals, rendering it the foremost contributor to hospitalizations attributable to arrhythmias. Typical manifestations of AF encompass palpitations, irregular cardiac rhythm, dyspnea, chest discomfort, and other related symptoms. However, it is noteworthy that AF can also manifest without overt symptoms, thereby denoted as silent AF (1, 2).

High-altitude areas are defined as regions situated at elevations of 2,700 meters or more above the mean sea level. These areas are characterized by lower air density and reduced oxygen availability (3, 4). Exposure to high altitude presents a notable stressor that affects cardiovascular function and autonomic regulation. Whether experienced briefly or for extended periods, being at high altitudes triggers dysfunction in the respiratory, cardiovascular, and endocrine systems. The development of cardiovascular diseases is influenced by various factors, with the hypoxic environment at high altitudes standing out as a significant contributor to increased cardiac strain (5–7). Hypoxia, which is characterized by insufficient oxygen supply, primarily triggers changes in the atria of the heart, often leading to atrial fibrosis. This alteration contributes to a higher incidence of AF, a commonly observed arrhythmia. AF arises due to cycles of intermittent hypoxia, disrupting the balance of cardiac autonomic modulation (8, 9).

The Knowledge, Attitude, and Practices (KAP) survey constitutes a research methodology aimed at evaluating the comprehension, perspectives, and behaviors of a particular group in relation to a specific subject matter, often applied in the realm of health literacy. This model hinges on the fundamental concept that knowledge exerts a positive influence on attitudes, subsequently shaping practices (10, 11). In the realm of AF research, understanding the knowledge, attitudes, and associated practices of the general population in high-altitude regions regarding AF can facilitate the development of tailored health education and intervention strategies, aimed at enhancing early detection, management, and prevention of AF. However, despite a few existing studies exploring KAP in the context of AF, these have predominantly centered around healthcare professionals and patients, with a notable absence of geographically tailored investigations (12–14). Thus, there remains a noticeable scarcity of research that delves into the KAP dimensions of AF among individuals residing in high-altitude regions, emphasizing the significant avenue for further exploration in this realm.

Therefore, this study aimed to investigate the KAP of AF among the general population in high-altitude regions.

## Methods

### Study design and participants

This cross-sectional study was conducted between July 2023 and August 2023 in high-altitude areas, involving the general population. Adults aged 18 years or older residing at high altitude were included, and written informed consent was required. In order to maximize participant inclusion, we initially set no exclusion criteria during the recruitment phase. However, to ensure the quality of the research, exclusions were subsequently made based on the data collected. The exclusion criteria applied were as follows: Participants under the age of 18, individuals who elected not to participate in the study, and respondents whose questionnaire completion time was less than 90 s (According to the pre-test for the questionnaire, the researchers found that those the answer time less than 90 s were more likely to have careless answers) were excluded from the study. This study was approved by the author’ Hospital and obtained informed consent from all participants.

### Questionnaires and quality control

A questionnaire consisting of four dimensions was formulated based on pertinent literature (15, 16). After creating the initial questionnaire, the opinions of two experts in the field of cardiovascular diseases, working at the author’ Hospital, were sought. Their recommendations were incorporated, leading to the removal of repetitive items and clarification of unclear questions. The final questionnaire encompassed the following dimensions: (1) Demographic information of participants, including age, gender, type of residence, education level, employment status, income level, and other 12 related questions. (2) Knowledge dimension, comprising 10 questions regarding atrial fibrillation. Scores ranged from 2 (very knowledgeable) to 0 (unaware), based on the level of knowledge. (3) Attitude dimension, consisting of 8 questions. A five-point Likert scale was used, ranging from 5 (very positive) to 1 (very negative). (4) Practice dimension, containing 9 questions. Similarly, a five-point Likert scale was employed, ranging from 5 (always) to 1 (never). Higher scores indicated better knowledge, more positive attitudes, and more proactive practices. Attaining scores above 70% of the maximum in each section indicated adequate knowledge, positive attitude, and proactive practice (17).

Before commencing the main survey, a pilot study was executed, involving 62 participants, to evaluate internal consistency. Utilizing Cronbach’s α coefficient, the analysis yielded a score of 0.852, signifying robust internal reliability. Subsequently, an online questionnaire was meticulously formulated using the Wen Juan Xing (WJX) platform.^1^ For data collection, a dedicated QR code was generated, facilitating seamless engagement through WeChat. A convenience sampling method was employed for participant recruitment. The researcher distributed the questionnaire through doctors familiar with the study in high altitude areas. These doctors then forwarded the questionnaire via WeChat group to the general population who were interested in the questionnaire’s topic. This method was chosen in an effort to extend the reach of the survey within high-altitude areas and maximize participant engagement. Participants accessed and completed the questionnaire by scanning the provided QR code via WeChat. Participants can enter the questionnaire only by checking the “informed consent” on the home page. All data were collected anonymously. To ensure the response quality and comprehensiveness, and prevent duplication, IP restriction was applied, allowing only one completion of the survey from a single IP address. To guarantee data integrity, internal coherence, and overall reasonability, the research team diligently scrutinized the amassed dataset.

### Statistical analysis

Statistical analysis was performed using Stata 17.0 (Stata Corporation, College Station, TX, USA). Quantitative variables were described as mean ± standard deviation (SD), and group comparisons were conducted using t-tests or analysis of variance (ANOVA). Categorical variables were presented as *n* (%). Pearson correlation analysis was used to explore the relationships between knowledge, attitude, and practice scores. Logistic regression was employed for both univariate and multivariate analyses of practice, using 70% of the total score as the cut-off value (18). The inclusion of variables in the multivariate regression model was based on the significance level (*p* < 0.05) observed in the univariate analysis. SEM was conducted to test the hypothesis that (H1) knowledge directly affects attitude; (H2) knowledge directly affects practice; and (H3) attitude directly affects practice. Two-sided *p* < 0.05 was considered statistically significant.

## Results

The study initially collected 902 questionnaires. Firstly, six participants who were under the age of 18 were identified and excluded. Subsequently, 30 individuals chose not to partake in the study, and finally, 111 respondents were removed due to their questionnaire completion time falling short of 90 s. This culminated in a total of 786 valid questionnaires that were ultimately utilized for the final analysis. Among them, 347 (44.15%) were male, with an average age of 34.75 ± 14.16 years. There were 262 (59.03%) participants resided in urban areas, and 161 (20.48%) participants were the Tibetan demographic. Approximately 353 participants (44.91%) held bachelor’s degrees, and 261 participants (33.21%) occupied managerial or professional/technical positions. In terms of employment, 516 participants (65.65%) were employed, with 262 participants (33.33%) reporting a monthly income ranging from 5,000 to 10,000 CNY. Marital status showed that 460 participants (58.52%) were married (Table 1).

**Table 1:** Demographic characteristics.

Variables	*N* (%)	Knowledge, Mean ± SD	*p*	Attitude, Mean ± SD	*p*	Practice, Mean ± SD	*p*
Total score	*N* = 786	8.22 ± 6.50		28.90 ± 5.63		34.34 ± 6.44	
Age, years	34.75 ± 14.16						
Gender			0.198		<0.001		<0.001
Male	347 (44.15)	7.88 ± 6.42		27.55 ± 5.65		32.89 ± 6.43	
Female	439 (55.85)	8.49 ± 6.55		29.97 ± 5.38		35.48 ± 6.21	
Residence			<0.001		<0.001		<0.001
Rural	262 (33.33)	6.85 ± 6.18		27.08 ± 5.93		32.63 ± 6.75	
City	464 (59.03)	8.91 ± 6.65		29.81 ± 5.19		35.12 ± 6.20	
Suburbs	60 (7.63)	8.88 ± 6.53		29.80 ± 5.70		35.78 ± 5.30	
Ethnicity			0.056		<0.001		<0.001
Tibetan	161 (20.48)	7.15 ± 6.70		26.66 ± 5.99		32.61 ± 7.01	
Han Chinese	536 (68.19)	8.55 ± 6.47		29.67 ± 5.40		34.97 ± 6.19	
Ethnic Minorities	89 (11.32)	8.17 ± 6.11		28.33 ± 5.18		33.64 ± 6.24	
Education			<0.001		<0.001		<0.001
Junior high school and below	134 (17.05)	6.19 ± 6.01		27.39 ± 5.13		31.65 ± 6.81	
High School/Technical Secondary School	100 (12.72)	6.36 ± 5.26		27.44 ± 5.85		33.25 ± 6.51	
College	158 (20.10)	7.91 ± 6.19		28.62 ± 5.45		34.25 ± 6.22	
Bachelor degree	353 (44.91)	9.53 ± 6.65		30.27 ± 5.39		35.88 ± 6.00	
Postgraduate and above	41 (5.22)	9.32 ± 7.75		26.68 ± 6.49		32.85 ± 5.91	
Occupation							
Managers and Professional/Technical Personnel	261 (33.21)	11.18 ± 6.94	<0.001	29.69 ± 5.77	0.004	35.93 ± 6.23	<0.001
General Staff	132 (16.79)	7.60 ± 5.51		28.48 ± 5.65		34.14 ± 5.89	
Service Industry and Production Workers	81 (10.31)	4.84 ± 5.60		27.20 ± 5.64		32.02 ± 6.92	
Others	312 (39.69)	6.88 ± 5.75		28.86 ± 5.40		33.69 ± 6.42	
Employment			0.100		0.053		0.619
Employed	516 (65.65)	8.50 ± 6.54		28.62 ± 5.67		34.26 ± 6.44	
Not Employed	270 (34.35)	7.69 ± 6.38		29.44 ± 5.52		34.50 ± 6.44	
Average Monthly Income, RMB			<0.001		0.003		<0.001
<2000	126 (16.03)	6.77 ± 6.37		27.41 ± 5.52		32.37 ± 6.70	
2000–5,000	236 (30.03)	7.80 ± 6.48		28.57 ± 5.69		34.14 ± 6.42	
5,000–10,000	262 (33.33)	8.60 ± 6.37		29.34 ± 5.33		34.78 ± 6.37	
10,000–20,000	109 (13.87)	10.35 ± 6.36		30.02 ± 5.63		36.04 ± 5.63	
>20,000	53 (6.74)	7.30 ± 6.71		29.45 ± 6.36		34.19 ± 6.74	
Maritial Status			0.255		0.012		0.151
Not married	326 (41.48)	8.53 ± 6.79		29.50 ± 5.84		34.73 ± 6.78	
Married	460 (58.52)	8.00 ± 6.27		28.48 ± 5.44		34.06 ± 6.17	
Presence of Underlying Health Conditions			0.398		<0.001		0.073
Yes	157 (19.97)	7.83 ± 5.69		27.32 ± 5.22		33.52 ± 6.18	
No	629 (80.03)	8.32 ± 6.68		29.29 ± 5.66		34.54 ± 6.49	
Smoking Habits			0.017		<0.001		<0.001
Never Smoked	581 (73.92)	8.60 ± 6.60		29.41 ± 5.59		35.16 ± 6.29	
Former Smoker	95 (12.09)	7.53 ± 5.95		28.29 ± 5.21		33.23 ± 6.06	
Current Smoker	110 (13.99)	6.82 ± 6.18		26.72 ± 5.65		30.97 ± 6.34	
Health insurance			<0.001		0.013		<0.001
Only one type of insurance	559 (71.12)	8.28 ± 6.45		28.99 ± 5.63		34.41 ± 6.41	
Two types of insurance	177 (22.52)	8.95 ± 6.46		29.25 ± 5.67		35.18 ± 6.17	
No insurance	50 (6.36)	4.92 ± 6.22		26.68 ± 5.07		30.58 ± 6.52	
Family History of AF			<0.001		0.006		0.233
Yes	82 (10.43)	10.89 ± 5.35		27.29 ± 5.71		33.54 ± 6.32	
No	704 (89.57)	7.91 ± 6.55		29.09 ± 5.59		34.43 ± 6.45	

The mean scores for knowledge, attitude, and practice were 8.22 ± 6.50 (possible range: 0–10), 28.90 ± 5.63 (possible range: 8–40), and 34.34 ± 6.44 (possible range: 9–45), respectively. The study reveals significant gender-related impacts on attitude (*p* < 0.001) and practice (*p* < 0.001) scores, along with notable variations based on residence (knowledge: *p* < 0.001, attitude: *p* < 0.001, practice: *p* < 0.001), indicating regional differences. Education strongly influences all three domains (knowledge: *p* < 0.001, attitude: *p* < 0.001, practice: *p* < 0.001), while occupation highlights noteworthy disparities among managers and professional/technical personnel in knowledge (*p* < 0.001), attitude (*p* = 0.004), and practice (*p* < 0.001) scores. Monthly income correlates with knowledge (*p* < 0.001) and practice (*p* < 0.001) scores, smoking habits associate significantly (knowledge: *p* = 0.017, attitude: *p* < 0.001, practice: *p* < 0.001), health insurance relates to all dimensions (knowledge: *p* < 0.001, attitude: *p* = 0.013, practice: *p* < 0.001), and a family history of AF links to knowledge (*p* < 0.001) and attitude (*p* = 0.006) scores (Table 1).

In the knowledge dimension, the three questions with the highest proportion of participants selecting the “Very familiar” option were as follows: the question regarding “Preventing AF necessitates addressing underlying conditions” (K10), chosen by 21.37% of respondents; the question about “Thrombus formation and subsequent embolization present the gravest risks associated with AF” (K4), selected by 20.61%; and the question on “Atrial fibrillation, commonly referred to as AF or ‘atrial flutter’“(K1), chosen by 20.36%. Conversely, the three questions with the highest percentage of participants selecting the “Unfamiliar” option were: the question discussing “Preventing AF necessitates addressing underlying conditions” (K9), with 46.44% choosing this option; the question related to “Catheter ablation (radiofrequency ablation) currently stands as the sole curative treatment for AF” (K8), chosen by 42.37%; and the question about “Effective anticoagulation therapy plays a crucial role in preventing and reducing AF-related stroke and thromboembolic events” (K7), selected by 42.24% (Table 2).

**Table 2:** Knowledge, attitude, and practice.

Statement	Very familiar	Familiar	Unfamiliar
1.Atrial fibrillation, commonly referred to as AF or “atrial flutter,” represents one of the most prevalent cardiac arrhythmias. In layman’s terms, it manifests as irregular contractions of the atria, particularly the left atrium, due to disordered electrical signals. This chaotic activity leads to uncoordinated contractions of different atrial sections, resulting in an irregular and possibly detrimental impact on the overall heart rhythm.	160 (20.36)	367 (46.69)	259 (32.95)
2.AF not only impairs patients’ quality of life but can also lead to severe complications such as thromboembolism and heart failure. The most critical complication associated with AF is stroke, also known as cerebrovascular accident (CVA) or cerebral infarction.	156 (19.85)	385 (48.98)	245 (31.17)
3.Clinical manifestations of AF vary widely. Most patients experience symptoms such as palpitations, chest discomfort, fatigue, dizziness, and weakness. However, some individuals remain asymptomatic, and the condition may only be detected when serious complications like stroke or heart failure occur.	151 (19.21)	382 (48.60)	253 (32.19)
4.Thrombus formation and subsequent embolization present the gravest risks associated with AF. In this condition, the loss of atrial contractile function leads to blood stasis within the atria, resulting in thrombus formation. Detachment of these clots can lead to embolism, causing cerebral embolism (stroke, hemiplegia), peripheral arterial embolism (in severe cases, even requiring amputation), and others.	162 (20.61)	370 (47.07)	254 (32.32)
5.The diagnosis of AF relies on a combination of medical history, physical examination, and electrocardiogram (ECG) assessment. The evaluation begins with gathering information on clinical manifestations like chest discomfort and palpitations. Subsequently, the physical examination helps ascertain any variations in heart sounds, irregular heart rhythm (heart irregularity), or pulse deficits (pulse rate lower than heart rate). If any of these indications are present, an ECG should be conducted for a definitive diagnosis.	159 (20.23)	350 (44.53)	277 (35.24)
6.Treatment approaches for AF encompass various options, including pharmacotherapy, electrical cardioversion, radiofrequency ablation, and surgical interventions. The selection of treatment depends on the patient’s disease progression, severity, and individual preferences.	144 (18.32)	351 (44.66)	291 (37.02)
7.Effective anticoagulation therapy (e.g., with medications such as warfarin, dabigatran, or rivaroxaban) plays a crucial role in preventing and reducing AF-related stroke and thromboembolic events.	145 (18.45)	309 (39.31)	332 (42.24)
8.Catheter ablation (radiofrequency ablation) currently stands as the sole curative treatment for AF, applicable to the vast majority of AF patients, especially those in the early stages of the disease with limited cardiac structural abnormalities. This minimally invasive procedure boasts high success rates and fast recovery times.	132 (16.79)	321 (40.84)	333 (42.37)
9.AF patients are at an elevated risk of experiencing stroke. To prevent AF-related strokes in patients with a high bleeding risk from anticoagulant medications or those unwilling or unable to adhere to long-term oral anticoagulant therapy, doctors may recommend left atrial appendage occlusion (closure) surgery.	118 (15.01)	303 (38.55)	365 (46.44)
10.Preventing AF necessitates addressing underlying conditions (e.g., heart disease, hyperthyroidism) proactively and adopting a healthy lifestyle, including smoking cessation, limited alcohol consumption, and maintaining a healthy weight.	168 (21.37)	333 (42.37)	285 (36.26)

Attitude	Strongly Agree	Agree	Neutral	Disagree	Strongly Disagree
1.I am aware of the dangers of atrial fibrillation (AF) and, therefore, am willing to actively follow the advice of medical professionals to improve my health. [P]	357 (45.42)	330 (41.98)	65 (8.27)	15 (1.91)	19 (2.42)
2.I believe AF is not severe and does not require treatment. I consider it normal to have such conditions at an older age, and I think it can be self-resolving or managed with traditional methods alone. [N]	64 (8.14)	122 (15.52)	87 (11.07)	302 (38.42)	211 (26.84)
3.I feel fearful or concerned about AF, worrying that it may lead to serious health issues. [N]	139 (17.68)	321 (40.84)	239 (30.41)	65 (8.27)	22 (2.80)
4.I hope to receive more educational information about AF from healthcare professionals. [P]	313 (39.82)	364 (46.31)	78 (9.92)	17 (2.16)	14 (1.78)
5.I am indifferent to the existence of AF, thinking that it only affects people with underlying health conditions, and I believe I cannot develop AF. [N]	54 (6.87)	107 (13.61)	115 (14.63)	332 (42.24)	178 (22.65)
6.The medical facilities in our area are average, and going to a large hospital for treatment is troublesome. Consequently, I am not willing to dwell on it. [N]	56 (7.12)	123 (15.65)	143 (18.19)	310 (39.44)	154 (19.59)
7.Even if I were to be diagnosed with AF, I do not believe I have sufficient financial means to handle it, or I am not willing to spend that much money on treatment. [N]	53 (6.74)	116 (14.76)	132 (16.79)	336 (42.75)	149 (18.96)
8.I think I am fine as long as I do not experience any symptoms, and I believe doctors are just trying to scare me. [N]	38 (4.83)	70 (8.91)	91 (11.58)	364 (46.31)	223 (28.37)

Practice	Always	Frequently	Sometimes	Occasionally	Never
1.I will actively and proactively monitor my health status, promptly recognizing symptoms of atrial fibrillation (AF), and seek medical attention proactively. [P]	208 (26.46)	229 (29.13)	204 (25.95)	113 (14.38)	32 (4.07)
2.I will adhere to medical advice and undergo regular medical examinations as recommended. [P]	230 (29.26)	258 (32.82)	175 (22.26)	92 (11.7)	31 (3.94)
3.I will cooperate with the doctor’s recommendations and take proactive measures to manage any underlying conditions. [P]	262 (33.33)	278 (35.37)	163 (20.74)	62 (7.89)	21 (2.67)
4.I may mistakenly perceive AF symptoms as common “palpitations” and thus resort to inappropriate self-medication. [N]	74 (9.41)	101 (12.85)	156 (19.85)	135 (17.18)	320 (40.71)
5.Instead of visiting a hospital, I am more likely to explore various folk remedies that I personally consider effective. [N]	48 (6.11)	86 (10.94)	116 (14.76)	156 (19.85)	380 (48.35)
6.Adopting a prudent lifestyle to reduce the risk of AF occurrence includes the following practices: [P]					
6.1.Quitting smoking and limiting alcohol consumption	405 (51.53)	146 (18.58)	93 (11.83)	74 (9.41)	68 (8.65)
6.2.Prioritizing psychological well-being	354 (45.04)	226 (28.75)	125 (15.9)	56 (7.12)	25 (3.18)
6.3.Maintaining a balanced diet	298 (37.91)	223 (28.37)	170 (21.63)	75 (9.54)	20 (2.54)
6.4.Engaging in moderate physical activity	246 (31.3)	197 (25.06)	186 (23.66)	125 (15.9)	32 (4.07)

AF, atrial fibrillation; CVA, cerebrovascular accident; ECG, electrocardiogram; P, positive; N, negative.

In the attitude dimension, a significant 40.84% of participants expressed their concerns and apprehensions regarding AF, fearing its potential escalation into serious health complications (A3). Furthermore, a noteworthy 39.82% conveyed their desire for more comprehensive educational resources concerning AF, highlighting the demand for improved information dissemination by healthcare professionals (A4). Conversely, a notable proportion (42.24%) displayed a degree of indifference towards AF’s existence, believing it exclusively affects individuals with underlying health conditions and dismissing their susceptibility to AF development (A5). Equally relevant is the observation that a substantial segment (39.44%) exhibited reluctance to engage with AF due to concerns over medical facility quality and the inconvenience of accessing specialized care (A6). Additionally, a significant percentage (46.31%) expressed their belief that they remain unaffected as long as symptomatic manifestations are absent, attributing doctors’ warnings to exaggeration rather than genuine medical concern (A8). Finally, a notable proportion (42.75%) expressed reservations about the financial feasibility of managing AF or their willingness to allocate substantial resources to its treatment (A7) (Table 2).

In the practice dimension, it is noteworthy that 26.46% of patients demonstrate a commitment to actively monitor their health for AF symptoms (P1), while 29.26% express dedication to adhering to medical advice and recommended examinations (P2). Furthermore, 33.33% of participants assert their intent to cooperate with doctors’ recommendations for managing underlying conditions (P3). On the other hand, attention is drawn to the potential for symptom misinterpretation and subsequent inappropriate self-medication, acknowledged by 17.18% of patients (P4). Additionally, 6.11% of patients exhibit a preference for alternative remedies over hospital visits (P5). Lifestyle practices concerning AF risk reduction also display varying levels of commitment: 51.53% prioritize quitting smoking and limiting alcohol (P6.1), 45.04% emphasize psychological well-being (P6.2), 37.91% commit to maintaining a balanced diet (P6.3), and 31.3% endorse engagement in moderate physical activity (P6.4) (Table 2).

Pearson correlation analysis revealed positive correlations between knowledge and attitude (*r* = 0.125, *p* < 0.001), knowledge and practice (*r* = 0.327, *p* < 0.001), and attitude and practice (*r* = 0.503, *p* < 0.001) (Supplementary Table S1).

Multivariate logistic regression analysis demonstrated that knowledge scores (OR = 1.108, 95% CI = 1.075–1.142, *p* < 0.001), attitude scores (OR = 1.118, 95% CI = 1.081–1.156, *p* < 0.001), and never smoking (OR = 2.438, 95% CI = 1.426–4.167, *p* = 0.001) were independently associated with practice (Table 3).

**Table 3:** Univariate and multivariate logistic regression analysis for proactive practice.

Practice Dimension	Univariate logistic regression		Multivariate logistic regression	
	OR (95% CI)	*p*	OR (95% CI)	*p*
Knowledge scores	1.110 (1.080–1.139)	<0.001	1.108 (1.075–1.142)	<0.001
Attitude scores	1.122 (1.090–1.154)	<0.001	1.118 (1.081–1.156)	<0.001
Gender				
Male	1.522 (1.127–2.056)	0.006	1.296 (0.846–1.985)	0.233
Female	ref		ref	
Age, years	0.991 (0.981–1.001)	0.081		
Residence				
Rural	ref		ref	
City	1.869 (1.360–2.567)	<0.001	0.999 (0.657–1.519)	0.996
Suburbs	2.940 (1.492–5.795)	0.002	1.713 (0.802–3.655)	0.164
Ethnicity				
Tibetan	0.571 (0.396–0.822)	0.003	0.946 (0.613–1.458)	0.800
Han Chinese	ref		ref	
Ethnic Minorities	0.725 (0.452–1.161)	0.181	0.890 (0.517–1.530)	0.673
Education				
Junior high school and below	0.890 (0.441–1.794)	0.744	1.224 (0.512–2.929)	0.649
High School/Technical Secondary School	1.753 (0.835–3.682)	0.138	2.593 (1.054–6.376)	0.038
College	1.663 (0.829–3.337)	0.152	1.700 (0.738–3.914)	0.212
Bachelor degree	2.854 (1.473–5.532)	0.002	2.121 (0.964–4.666)	0.062
Postgraduate and above	ref		ref	
Occupation				
Managers and Professional/Technical Personnel	1.664 (1.153–2.402)	0.007	0.979 (0.622–1.543)	0.928
General Staff	0.957 (0.625–1.466)	0.841	0.895 (0.550–1.455)	0.653
Service Industry and Production Workers	0.662 (0.403–1.087)	0.103	0.918 (0.520–1.618)	0.767
Others	ref		ref	
Employment Status				
Employed	ref			
Not Employed	1.146 (0.835–1.575)	0.399		
Average Monthly Income, CNY				
<2000	0.676 (0.349–1.311)	0.247		
2000–5,000	1.325 (0.708–2.481)	0.379		
5,000–10,000	1.184 (0.638–2.198)	0.592		
10,000–20,000	1.979 (0.962–4.072)	0.064		
>20,000	ref			
Maritial Status				
Not married	1.223 (0.901–1.661)	0.197		
Married	ref			
Presence of Underlying Health Conditions				
Yes	1.125 (0.777–1.627)	0.534		
No	ref			
Smoking Habits				
Never Smoked	2.758 (1.820–4.179)	<0.001	2.438 (1.426–4.167)	0.001
Former Smoker	1.930 (1.100–3.385)	0.022	1.727 (0.930–3.210)	0.084
Current Smoker	ref		ref	
Health insurance				
Only one type of insurance	2.702 (1.504–4.855)	0.001	1.682 (0.868–3.261)	0.124
Two types of insurance	3.520 (1.837–6.746)	<0.001	1.969 (0.922–4.207)	0.080
No insurance	ref		ref	
Family History of AF				
Yes	0.969 (0.596–1.578)	0.900		
No	ref			

**Figure 1:**
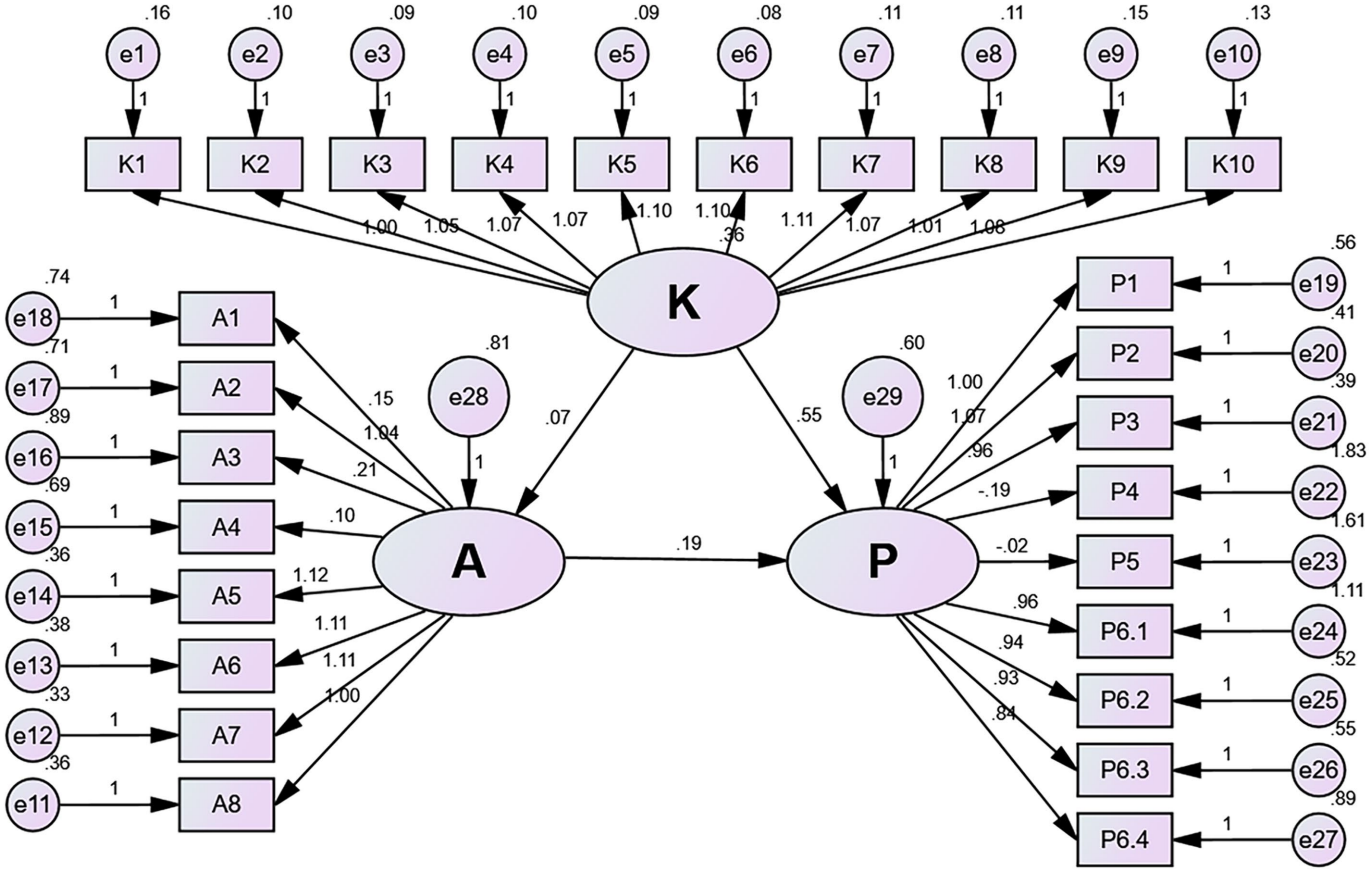
Structural equation modeling.

The SEM illustrated direct effect of knowledge on practice (*β* = 0.396, 95% CI = 0.331 ~ 0.464, *p* = 0.014), and attitude on practice (*β* = 0.216, 95% CI = 0.140 ~ 0.317, *p* = 0.004), while no effect of knowledge on attitude (*β* = 0.050, 95% CI = −0.036 ~ 0.118, *p* = 0.190). In addition, attitude showed no indirect effect between knowledge and practice (*β* = 0.011, 95% CI = −0.004 ~ 0.036, *p* = 0.117) (Figure 1, Supplementary Table S3). Supplementary Table S2 attest to the adequacy of the model fit.

## Discussion

The study’s results indicate that individuals living in high-altitude regions have sufficient knowledge, a positive attitude, and proactive practices related to AF (atrial fibrillation). In light of these findings, the adoption of effective strategies is recommended to bolster AF management, reduce associated risks, and ultimately improve overall patient outcomes.

In the domain of knowledge, participants demonstrated varying familiarity levels with different aspects of AF. Notably, questions related to recognizing AF as “atrial flutter” and understanding the associated risks of thrombus formation received higher familiarity scores, indicating a stronger comprehension in these areas. However, there was limited familiarity with concepts such as “Catheter ablation as a curative treatment,” highlighting the need for focused educational interventions to enhance understanding of advanced treatment modalities for AF. Converting these insights into effective strategies for improving clinical practice requires a strategic approach. Addressing the diverse familiarity levels among participants through targeted educational initiatives can effectively bridge knowledge gaps and promote a well-informed patient population (1, 19, 20).

Attitudinal heterogeneity was evident within the participant cohort, giving rise to discernible emotional outlooks. Evident in a significant fraction of participants, apprehensions surrounding the progression of AF into severe health complications indicate a notable level of consciousness. This apprehension might plausibly act as a catalyst, propelling more proactive interactions with healthcare practitioners in the pursuit of efficacious AF management (21, 22). In contrast, the subset of participants displaying apathy towards AF accentuates the urgency of dispelling misconceptions and dismantling the stigma enveloping the ailment. This particular discovery underscores the exigency for campaigns that heighten awareness, elucidating AF’s potential ramifications among individuals devoid of pre-existing health conditions. The broad spectrum of attitudes, encompassing concerns regarding AF-related complications on one end and apathy or skepticism on the other, underscores the pivotal role of communication strategies (23, 24). Clinicians can harness this comprehension to formulate patient-centric narratives, tailored to resonate with diverse dispositions, thereby fostering heightened engagement and empowerment.

Shifting the focus to practices, participants demonstrated a blend of commendable proactive behaviors and areas potentially open to improvement. Evident in their commitment to health monitoring, adherence to medical advice, and adoption of risk-reducing actions is a positive inclination towards effectively managing AF. Conversely, a tendency to favor alternative remedies over conventional medical interventions, along with a subset associating well-being exclusively with the absence of symptoms, highlights gaps in comprehending the importance of evidence-based medical approaches (25–27). Guided by these observed practice trends, the enhancement of clinical practices might concentrate on fortifying proactive behaviors while concurrently dispelling prevalent misunderstandings. In our study, we emphasize the formulation of interventions that prioritize evidence-based medical management, as substantial research indicates its effectiveness in improving patient outcomes (28). This approach is aligned with the overarching objective of our research, which is to elevate patient outcomes. While alternative remedies can play a role in patient care, their efficacy and safety are often less well-documented in scientific literature.

The noteworthy and affirmative correlations identified among knowledge and attitude, knowledge and practice, and attitude and practice distinctly emphasize the inherent interconnectedness among these dimensions (10, 11). Individuals with a more profound understanding of AF tend to demonstrate more favorable attitudes and adopt healthier practices. This intrinsic interdependence accentuates the imperative for interventions that address not only knowledge augmentation but also the transformation of attitudes and the modification of behaviors (29, 30). The firmly established correlations among knowledge, attitudes, and practices serve to underscore the inseparable interplay within these realms. Consequently, clinical interventions must embrace a holistic methodology that concurrently addresses all three domains to ensure enduring enhancements.

The outcomes of the multivariate logistic regression analysis offer valuable insights into the independent factors that influence positive practices. Notably, knowledge and attitude emerge as central determinants, underscoring the pivotal roles of education and perception in shaping behavior. Additionally, the discovery that individuals who have never smoked are more prone to adopting positive practices underscores the significance of lifestyle adjustments in the management of AF (31–33). Upon meticulous examination of the multivariate logistic regression findings, the evident conclusion is that knowledge and attitude play pivotal roles in driving positive practices (34, 35). This revelation equips clinicians with the means to prioritize patient education and cultivate a supportive, informed milieu that nurtures constructive attitudes towards AF management. Converting these insights into tangible clinical strategies necessitates an integrative approach. The synergistic utilization of evidence-based educational initiatives, platforms for patient engagement, and precise communication strategies may collectively contribute to fostering enlightened and proactive patient behavior.

The results obtained through SEM vividly illustrate the intricate interplay among knowledge, attitudes, and practices. This structural framework seamlessly aligns with the principle that interventions should holistically encompass all three domains to effectuate comprehensive enhancements in AF management. Translating this conceptual understanding into clinical practice necessitates the formulation of interventions that not only educate patients but also cultivate a proactive mindset, fostering a commitment to the consistent application of evidence-based practices (36, 37).

This study had some limitations. Firstly, the reliance on a web-based survey may introduce selection bias, as individuals without internet access or those less inclined to participate in online surveys could be underrepresented. This could impact the generalizability of the findings to the entire population in high-altitude regions. Secondly, the study’s cross-sectional design limits the ability to establish causal relationships between knowledge, attitude, and practice scores, as well as their associations with demographic factors. Longitudinal or interventional designs would be necessary to better understand the temporal relationships and potential interventions. Additionally, the use of self-reported data, which is subject to recall and social desirability biases, might lead to inaccuracies in participants’ responses and consequently affect the validity of the results. Moreover, the study did not differentiate participants based on personal or familial disease experiences, heart disease and risk factors such as hypertension therapy, which could potentially influence KAP regarding AF. Also, the younger age profile of our participants may not fully represent the broader population, potentially limiting the applicability of our findings. Despite these limitations, this study makes a significant contribution by elucidating the relationship between KAP and AF among the general population in high-altitude areas. The use of SEM to quantitatively confirm the positive influence of higher knowledge levels on attitudes and practices towards AF is a notable strength. This is particularly important in understanding the unique health perspectives and behaviors of high-altitude populations. Furthermore, identifying the association between non-smoking status and proactive AF management practices highlights an important target for health interventions, contributing valuable insights for public health strategies in these communities.

In conclusion, this study showed that the general population residing in high-altitude regions possesses adequate knowledge, positive attitude, and proactive practice towards AF. Building upon these findings, potential strategies emerge for optimizing AF management, including tailored educational interventions to address knowledge gaps, community-based initiatives for regular screenings and risk awareness, and systemic changes to ensure equitable healthcare access. By embracing these strategies, stakeholders can collaboratively work towards enhancing AF management, mitigating risks, and ultimately advancing patient outcomes.

## Data availability statement

The original contributions presented in the study are included in the article/Supplementary material, further inquiries can be directed to the corresponding author.

## Ethics statement

The studies involving humans were approved by this study was approved by Chengdu 363 Hospital Affiliated to Southwest Medical University Ethics Committee (2023033) and obtained informed consent from all participants. The studies were conducted in accordance with the local legislation and institutional requirements. Written informed consent for participation in this study was provided by the participants’ legal guardians/next of kin.

## Author contributions

KL: Investigation, Project administration, Resources, Writing – original draft, Writing – review & editing, Conceptualization, Methodology. JL: Investigation, Project administration, Resources, Writing – original draft, Writing – review & editing, Data curation. YZ: Data curation, Investigation, Methodology, Project administration, Visualization, Writing – original draft, Writing – review & editing.

## Footnotes

^1^ https://www.wjx.cn
