# Supplementary material for: Knowledge, attitude, and practice of atrial fibrillation in high altitude areas
Source: Front Public Health. 2024 Apr 10;12:1322366. doi: 10.3389/fpubh.2024.1322366 (PMC11039834; doi:10.3389/fpubh.2024.1322366)
Supplement: Supplementary file 1 [file Table_1.docx]

# **Table S1** Pearson correlation analysis.

|  | Knowledge | Attitude | Practice |
| --- | --- | --- | --- |
| Knowledge | 1 |  |  |
| Attitude | 0.125(*P*<0.001) | 1 |  |
| Practice | 0.327(*P*<0.001) | 0.503(*P*<0.001) | 1 |

**Table S2** Model Fit

| **Model** | **Ref.** | **Measured results** |
| --- | --- | --- |
| **CMIN/DF** | 1-3 excellent，3-5 good | 11.217 |
| **RMSEA** | <0.08 good | 0.114 |
| **IFI** | >0.8 good | 0.819 |
| **TLI** | >0.8 good | 0.802 |
| **CFI** | >0.8 good | 0.819 |

**Table S3** Model paths

| Model paths | Standardized Total effects | | Standardized direct effects | | Standardized indirect effects | |
| --- | --- | --- | --- | --- | --- | --- |
|  | β (95%CI) | P | β (95%CI) | P | β (95%CI) | P |
| K→A | 0.050 (-0.036~0.118) | 0.190 | 0.050 (-0.036~0.118) | 0.190 |  |  |
| K→P | 0.407 (0.345~0.474) | **0.013** | 0.396 (0.331~0.464) | **0.014** |  |  |
| A→P | 0.216 (0.140~0.317) | **0.004** | 0.216 (0.140~0.317) | **0.004** |  |  |
| K→P |  |  |  |  | 0.011 (-0.004~0.036) | 0.117 |
